# Increased haemodynamic adrenergic load with isoflurane anaesthesia in type 2 diabetic and obese rats *in vivo*

**DOI:** 10.1186/s12933-014-0161-4

**Published:** 2014-12-10

**Authors:** Carol T Bussey, Anne E de Leeuw, Regis R Lamberts

**Affiliations:** HeartOtago, Department of Physiology, Otago School of Medical Sciences, University of Otago, PO Box 56, Dunedin, 9054 New Zealand

**Keywords:** Anaesthesia, Conscious, Haemodynamic, Type 2 diabetes, Obesity, *in vivo*

## Abstract

**Background:**

Increasing numbers of type 2 diabetic and obese patients with enhanced rates of cardiovascular complications require surgical interventions, however they have a higher incidence of perioperative haemodynamic complications, which has been linked to adrenergic dysfunction. Therefore, we aimed to determine how α- and β-adrenoceptor (AR)-mediated haemodynamic responses are affected by isoflurane anaesthesia in experimental type 2 diabetes and obesity *in vivo*.

**Methods:**

Sixteen-week old male Zucker type 2 Diabetic Fatty (ZDF) rats, Zucker Obese rats and their lean counterparts (*n* = 7-9 per group) were instrumented with radio telemeters to record blood pressure and heart rate and with vascular access ports for non-invasive intravenous drug delivery *in vivo*. Haemodynamic effects of α-AR (phenylephrine; 1-100 μg.kg^−1^) or β-AR (dobutamine; 2-120 μg.kg^−1^) stimulation were assessed under conscious and anaesthetised (isoflurane; 2%) conditions.

**Results:**

Vascular α-AR sensitivity was increased in both diabetic (non-diabetic 80 ± 3 vs. diabetic 95 ± 4 ΔmmHg at 100 μg.kg^−1^; *p* < 0.05) and obese (lean 65 ± 6 vs. obese 84 ± 6 ΔmmHg at 20 μg.kg^−1^; *p* < 0.05) conscious rats. Interestingly, anaesthesia exacerbated and prolonged the increased α-AR function in both diabetic and obese animals (non-diabetic 51 ± 1 vs. diabetic 68 ± 4 ΔmmHg, lean 61 ± 5 vs. obese 84 ± 2 ΔmmHg at 20 μg.kg^−1^; *p* < 0.05). Meanwhile, β-AR chronotropic sensitivity was reduced in conscious diabetic and obese rats (non-diabetic 58 ± 7 vs. diabetic 27 ± 8 Δbpm, lean 103 ± 12 vs. obese 61 ± 9 Δbpm at 15 μg.kg^−1^; *p* < 0.05). Anaesthesia normalised chronotropic β-AR responses, via either a limited reduction in obese (lean 51 ± 3 vs. obese 66 ± 5 Δbpm; NS at 15 μg.kg^−1^) or increased responses in diabetic animals (non-diabetic 49 ± 8 vs. diabetic 63 ± 8 Δbpm, at 15 μg.kg^−1^; NS at 15 μg.kg^−1^).

**Conclusions:**

Long term metabolic stress, such as during type 2 diabetes and obesity, alters α- and β-AR function, its dynamics and the interaction with isoflurane anaesthesia. During anaesthesia, enhanced α-AR sensitivity and normalised β-AR function may impair cardiovascular function in experimental type 2 diabetes and obesity.

## Background

The rapid expansion of the type 2 diabetes and obesity co-epidemics impacts heavily on cardiovascular health. One clinically important, but often overlooked, cardiovascular consequence is that diabetic and obese patients have increased requirements for surgical treatments. Following surgery they have longer hospital stays and poorer survival compared to non-diabetic and lean patients [1-3]. Importantly, patients with metabolic syndrome are subject to a higher incidence of perioperative haemodynamic complications, even for non-cardiac related surgeries, which most likely relates to changes in autonomic control of the cardiovascular system [2,4-6].

Long-term changes in metabolism, such as during the metabolic syndrome [7], are characterised by increased muscle sympathetic nerve activity [8,9] and increases in plasma (nor)epinephrine levels [10], suggesting overall central sympathetic over-activation [7]. Consequently, several *ex vivo* studies demonstrated decreased β-AR expression in [11-14] and reduced β-AR responsiveness of [14-17] the heart in diabetes, with similar reports in obesity [18-20]. Less attention has focused on α-ARs, with *ex vivo* studies or studies in anaesthetised rats variably reporting unchanged [16,21], impaired [22,23] or enhanced [24,25] α-AR activity in the metabolic syndrome.

While the long-term metabolic stress of diabetes and obesity leads to haemodynamic dysregulation [4,26], cardiovascular function is also acutely challenged in the perioperative setting [4,27]. Diabetes and obesity are both known to augment cardiovascular responses to anaesthetics [16,28,29]; and the well described cardioprotective effects of volatile anaesthetics are reduced under conditions of metabolic stress [30]. For instance, sevoflurane elicited greater impairments in myocardial blood flow in a pilot study of type 2 diabetic patients [31]. Isoflurane anaesthesia also impairs baroreflex responsiveness [32], and obesity is associated with impaired baroreflex sympatho-inhibition [33]; both effects which are thought to be mediated via augmentation of central nervous system pathways. Furthermore, Amour *et al*. [16] showed in isolated papillary muscles that type 1 diabetes attenuated the potentiation of α- and β-AR responses by halogenated anaesthetics, suggesting an interaction between anaesthetic and metabolic-mediated α- and β-AR dysfunction. However, this approach does not address the peripheral and neural effects present *in vivo*, nor examine changes in type 2 diabetes. Furthermore, the lack of *in vivo* data under conscious conditions, limits the interpretation of anaesthetic effects on α- and β-AR function.

Therefore, the present study aimed to assess how isoflurane anaesthesia affects the α- and β-AR-mediated haemodynamic responses in type 2 diabetes *in vivo*. To assess the direct effects of anaesthesia we measured haemodynamic responses in free-moving conscious and in isoflurane anaesthetised type 2 diabetic (Zucker Diabetic Fatty (ZDF)) rats following α- and β-AR stimulation. To this end, rats were implanted with a radio telemetric transmitter and a vascular access port to measure *in vivo* abdominal aortic blood pressure and inject intravenous drugs, respectively [34]. These measures were repeated in obese (Zucker) rats to determine whether the observed changes were a specific effect of type 2 diabetes, or a more general feature of metabolic syndrome.

## Materials and methods

### Animals

All procedures were approved by the University of Otago Animal Ethics Committee and were conducted in accordance with the New Zealand Animal Welfare Act (1999). Zucker Diabetic Fatty (ZDF) rats are derived from a selected subset of the Zucker strain, which spontaneously develop diabetes from 12 weeks of age due to impaired pancreatic beta-cell function [35]. Zucker Obese rats have a homozygous missense mutation in the leptin receptor gene (fa/fa) leading to impaired satiety signalling and hyperphagia [36,37]. These strains are well-accepted models of type 2 diabetes mellitus and obesity, respectively, and were compared to their own lean littermates as in-strain controls.

**Figure 1:**
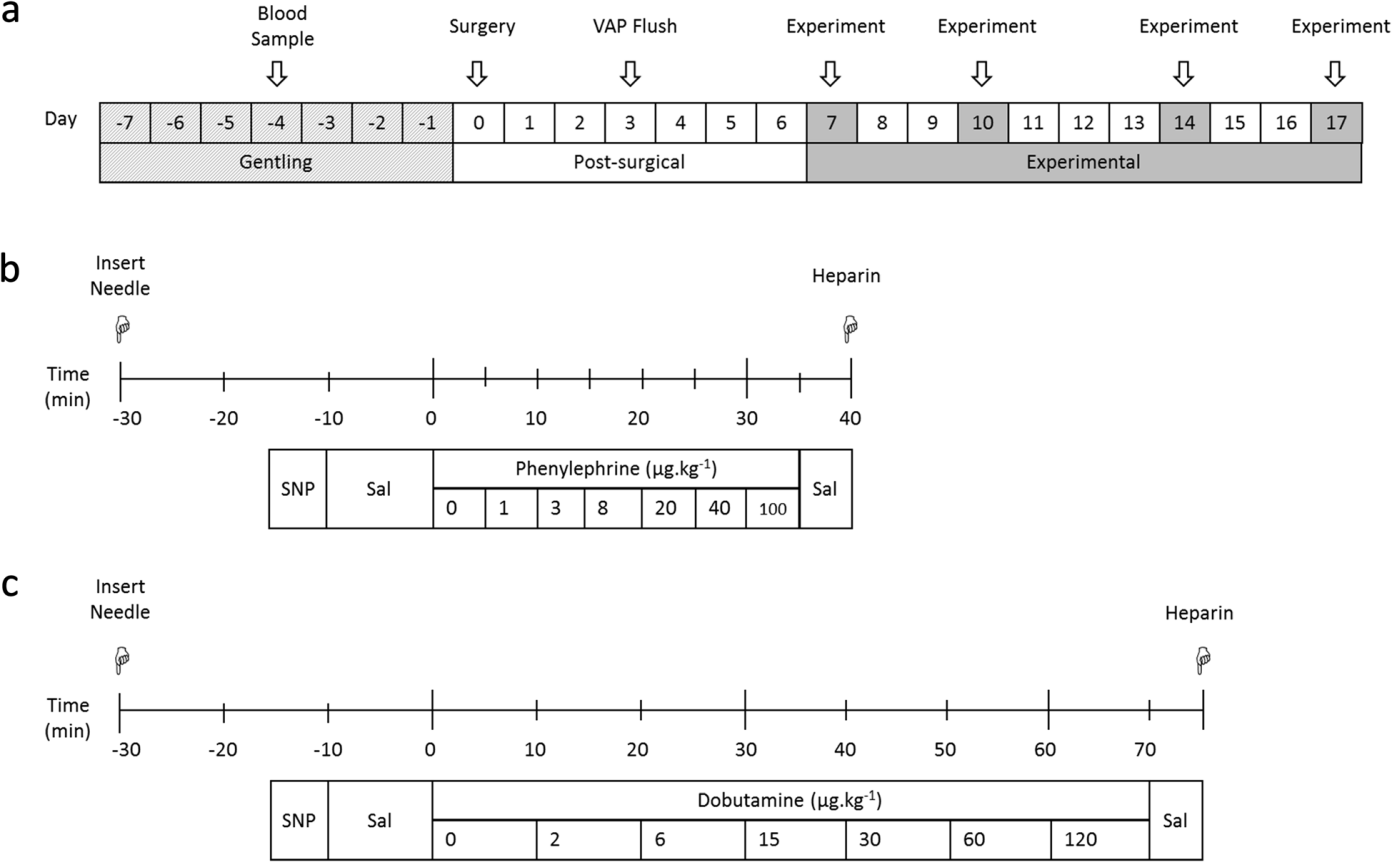
**Experimental protocols.** Overview of the study, indicating one week gentling and post-surgical recovery periods, and twice-weekly experimental sessions for randomised protocols **(a)**. Protocols for the acute administration of incrementing doses of the α-adrenergic agonist phenylephrine at five minute intervals **(b)**, and the β-adrenergic agonist dobutamine at ten minute intervals **(c)**. Sodium nitroprusside (SNP) was injected at the initiation of each experiment to confirm vascular access port (VAP) patency. Sal represents saline injection to flush the VAP. All solutions were administered as a bolus.

Male rats (*N* = 39; Charles River Laboratories, Wilmington, MA, USA) were housed at 20 ± 1°C under a 12 hour light–dark cycle and provided with food and water ad libitum. All ZDF animals were maintained on Purina 5008 diet (LabDiet®, St Louis, MO, USA) as recommended by the supplier, while Zucker rats were fed chow (Rat and Mouse cubes; Specialty Feeds, WA, Australia). Animals were gentled daily for one week prior to surgery (Figure 1a). Plasma samples were collected via the tail vein following an 8-hour fast 4 days prior to surgery. Plasma glucose concentrations were determined using a glucometer (Roche, Basel, Switzerland) and insulin was measured by ELISA (Millipore, Billerica, MA, USA).

### Surgical procedures

Dual implantation of a radio telemeter and vascular access port was performed on 16-week old animals under 2–2.5% isoflurane anaesthesia (Minrad Inc, Bethlehem, PA, USA) as previously described [34] with strict adherence to aseptic procedures. Analgesia (5 mg.kg^−1^ carprofen; Norbrook, Newry, Northern Ireland) and antibiotic (30 mg.kg^−1^ trimethoprim and sulphamethazine; Virbac, Carros, France) were administered subcutaneously.

Vascular Access Ports (VAP™; ROP-3H, hydromer-coated polyurethane, 3Fr; Access Technologies, Skokie, IL, USA) were primed with heparin sodium (100 IU.mL^−1^; Hospira Australia, Mulgrave, Australia). The VAP reservoir was secured on the back of the animal between the scapulae, with the VAP cannula tunnelled subcutaneously to the femoral vein. A radio telemeter with pressure sensitive tip (TRM53P; Telemetry Research, Millar Instruments, Houston, TX, USA) was implanted into the abdominal aorta. Animals were allowed one-week post-surgical recovery before experimentation commenced (Figure 1a). The VAP was flushed with 0.4 mL heparin sodium (100 IU.mL^−1^) at minimum twice-weekly to maintain patency.

### Experimental procedures

Experiments were performed in random order twice-weekly to reduce stress to the animals, as well as ensuring complete drug clearance and avoiding potential desensitisation of adrenoceptors. This 3–4 day allowance between randomised experimental sessions, along with the 7 day post-surgical recovery period, also served to minimise potential effects of repeated exposure to volatile anaesthetics, generally described as lasting 24–72 hours [38]. VAPs were accessed under strict aseptic conditions using a Huber point needle (PG24-625; Access Technologies, Skokie, IL, USA), following application of a short-acting local analgesic (5% lignocaine/prilocaine; AstraZeneca, North Ryde, NSW, Australia).

Haemodynamic measures were equilibrated for 15 minutes following restraint and needle insertion. The nitric oxide donor sodium nitroprusside (SNP; 6.25 μg.kg^−1^) was administered at the beginning of each experimental session, as used previously to confirm VAP patency over the course of the study [34]. Isoflurane is a volatile anaesthetic commonly used in the clinical setting, due to its minimal haemodynamic effects and rapid recovery times. For measures under anaesthetised conditions, induction was undertaken at 5% isoflurane with maintenance at 2% isoflurane. Incrementing doses of the α-adrenergic agonist phenylephrine (1-100 μg.kg^−1^) and β-adrenergic agonist dobutamine (2-120 μg.kg^−1^) were administered at five or ten minute intervals, respectively (Figure 1b and c). Any access via the VAP was concluded by injection of 0.4 mL Heparin sodium (100 IU.mL^−1^) to prevent coagulation. All chemicals were from Sigma-Aldrich (St Louis, MO, USA) and diluted in saline (0.9% NaCl; Baxter, Toongabbie, Australia) unless otherwise stated.

### Data and statistical analysis

Blood pressure data was derived from the telemeter according to the manufacturer’s instructions, and acquired using LabChart® 7 software (ADInstruments, Dunedin, New Zealand). Heart rate (HR) and mean arterial pressure (MAP) were derived from blood pressure recordings using the LabChart® blood pressure module, and averaged over every ten consecutive cycles. Basal haemodynamic data for an individual animal were averaged over up to four conscious replicates and up to two replicate anaesthetised measures. Haemodynamic responses were assessed as the calculated change between the peak response to and the baseline immediately preceding each individual bolus injection. Time course dynamics were assessed over 5 second averages. Statistical analysis was performed for baseline characteristics via t-test, or Mann–Whitney rank sums test where the assumptions were not met; or via two-way repeated measures ANOVA for all haemodynamic data. Differences between groups were identified using Student-Newman-Keuls post-hoc analysis (Sigmaplot™ 12.0, Systat Software Inc, Chicago, IL, USA) and significance assumed at the level of *p* < 0.05. Data are expressed as mean ± standard error of the mean (SE).

## Results

### Baseline characteristics and haemodynamics of type 2 diabetic rats

Type 2 diabetic rats exhibited body weights 25% higher than their non-diabetic littermates (Table 1). This was accompanied by significantly greater abdominal adiposity, as indicated by epididymal fat pad weight. Type 2 diabetic animals also exhibited both hyperglycaemia and hyperinsulinemia, characteristic of the condition.

**Table 1:** **Baseline characteristics and haemodynamics**

	**Non-Diabetic**	**Diabetic**	**Lean**	**Obese**
Body weight (g)	333 ± 6	417 ± 14*	364 ± 7	584 ± 16*
Epididymal fat weight (g)	1.45 ± 0.16	6.38 ± 0.67*	2.77 ± 0.29	17.57 ± 1.04*
Epididymal fat/Tibia length (g.cm^−1^)	0.40 ± 0.06	1.92 ± 0.18*	0.65 ± 0.07	4.57 ± 0.31*
Heart weight (g)	1.49 ± 0.06	1.52 ± 0.06	1.37 ± 0.06	1.56 ± 0.08
Heart weight/Tibia length (g.cm^−1^)	0.41 ± 0.02	0.44 ± 0.02	0.32 ± 0.02	0.40 ± 0.02*
Fasting plasma glucose (mmol.L^−1^)	6.4 ± 0.4	19.5 ± 4.4*	6.6 ± 0.3	7.1 ± 0.8
Fasting plasma insulin (ng.mL^−1^)	1.2 ± 0.3	8.0 ± 2.2*	1.1 ± 0.4	12.8 ± 3.2*
Mean arterial pressure (mmHg)				
Conscious	110.2 ± 4.7	114.4 ± 3.5	106.2 ± 4.7	125.9 ± 5.5*
Anaesthetised	87.5 ± 4.4^†^	90.8 ± 7.6^†^	86.1 ± 5.8^†^	111.2 ± 5.0*^†^
Heart rate (bpm)				
Conscious	366 ± 16	317 ± 11*	389 ± 10	391 ± 10
Anaesthetised	314 ± 10^†^	292 ± 4^†^	365 ± 7^†^	365 ± 6^†^

Baseline characteristics of 16 week old Zucker Diabetic Fatty (ZDF) and Zucker Obese rats and their lean littermates. *significantly different from control littermates, ^†^significantly different anaesthetised vs. conscious, *n* = 6-9, *p* < 0.05, values are means ± SE.

Baseline haemodynamics were assessed under conscious resting conditions or following stabilisation of 2% isoflurane anaesthesia (Table 1). Type 2 diabetic animals were normotensive with a markedly reduced HR under conscious conditions; in agreement with multiple literature reports [10,39]. Isoflurane anaesthesia significantly reduced both HR and MAP in all animals, eliminating differences in HR.

### α-adrenergic sensitivity is increased in conscious and anaesthetised type 2 diabetic rats

**Figure 2:**
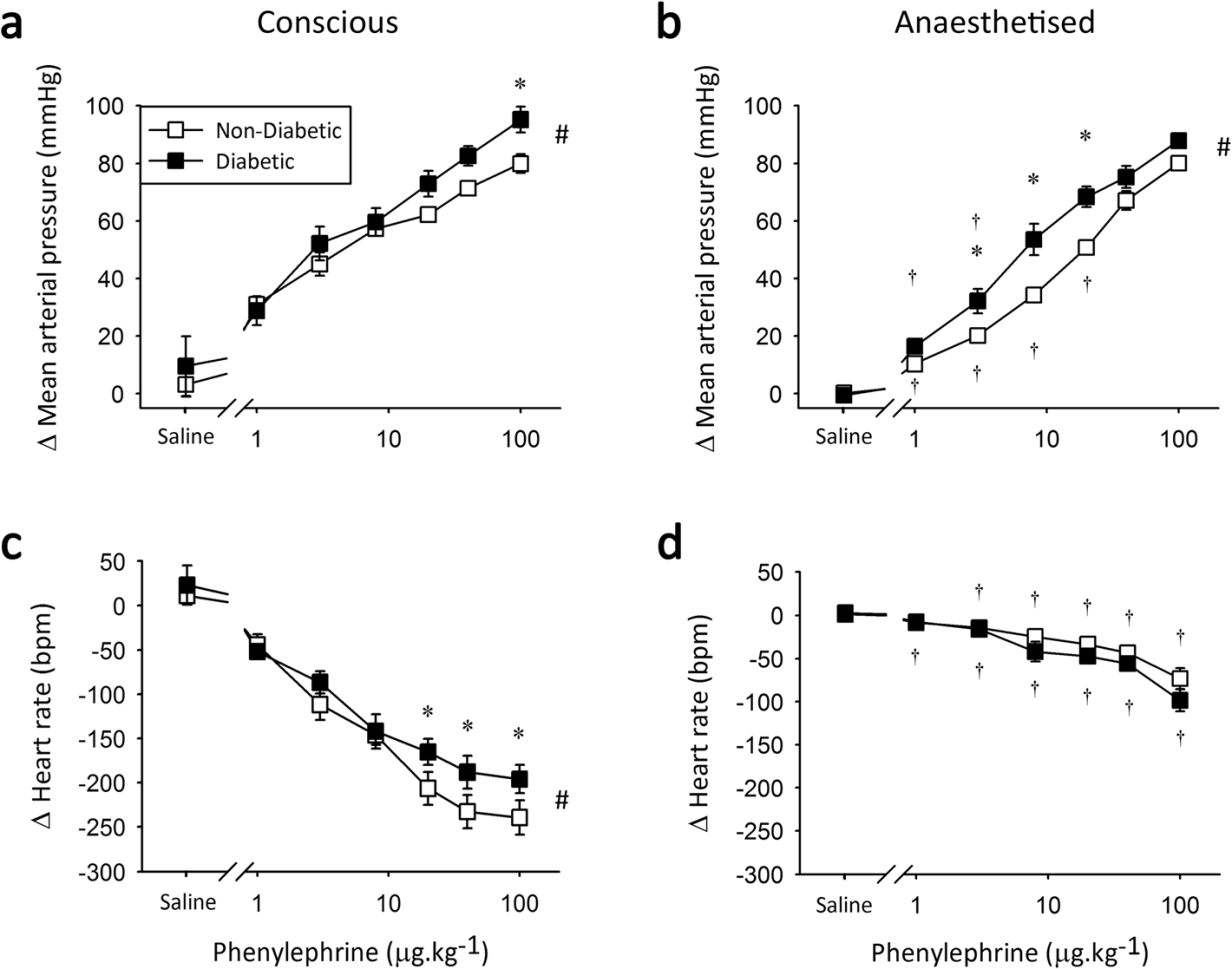
**α-adrenoceptor agonist responses in Zucker Diabetic Fatty rats.** Peak change in mean arterial pressure (top) and heart rate (bottom) in response to 1–100 μg.kg^−1^ phenylephrine under conscious conditions **(a and c)** and during isoflurane anaesthesia **(b and d)**. *significantly different from non-diabetic littermate controls, ^†^significantly different from conscious measure and # significant overall group difference, *n* = 8, *p* < 0.05, values are means ± SE.

Administration of phenylephrine (PE), an α-AR agonist, primarily elicited a rapid, transient increase in MAP in a dose-dependent manner (Figure 2). Conscious type 2 diabetic animals demonstrated a greater increase in MAP with the highest dose of phenylephrine (Figure 2a). It is unclear whether this represents maximal PE-mediated vasoconstriction, as the extreme bradycardia (Figure 2c) prevented testing of higher doses. However, higher doses of PE are unlikely to be used clinically, with a therapeutic dose range for PE of approximately 0.5-9ug.kg^−1^.min^−1^ [40]. Under anaesthesia the maximal MAP response to α-AR stimulation was not changed, however the increase in MAP at lower, therapeutic doses of PE was significantly reduced in both groups (Figure 2b). This isoflurane-induced decrease in pressure was less in the type 2 diabetic rats, accentuating the differential α-AR sensitivity in diabetes *in vivo*.

Secondary to the changes in MAP, PE elicited a dose-dependent reduction in HR (Figure 2c and d). Conscious diabetic animals experienced a reduced bradycardic response to high-dose PE (Figure 2c). Given the greater maximum recorded peak MAP (Figure 2a), this indicates a limited baroreflex capacity in conscious type 2 diabetic rats (diabetic −2.1 ± 0.2 bpm.mmHg^−1^ vs. non-diabetic −3.0 ± 0.3 bpm.mmHg^−1^ at 100 μg.kg^−1^; *p* < 0.05). Anaesthesia markedly reduced the baroreflex-mediated bradycardia in all animals, eliminating the between group differences.

### β-adrenergic sensitivity is reduced in conscious type 2 diabetic rats

**Figure 3:**
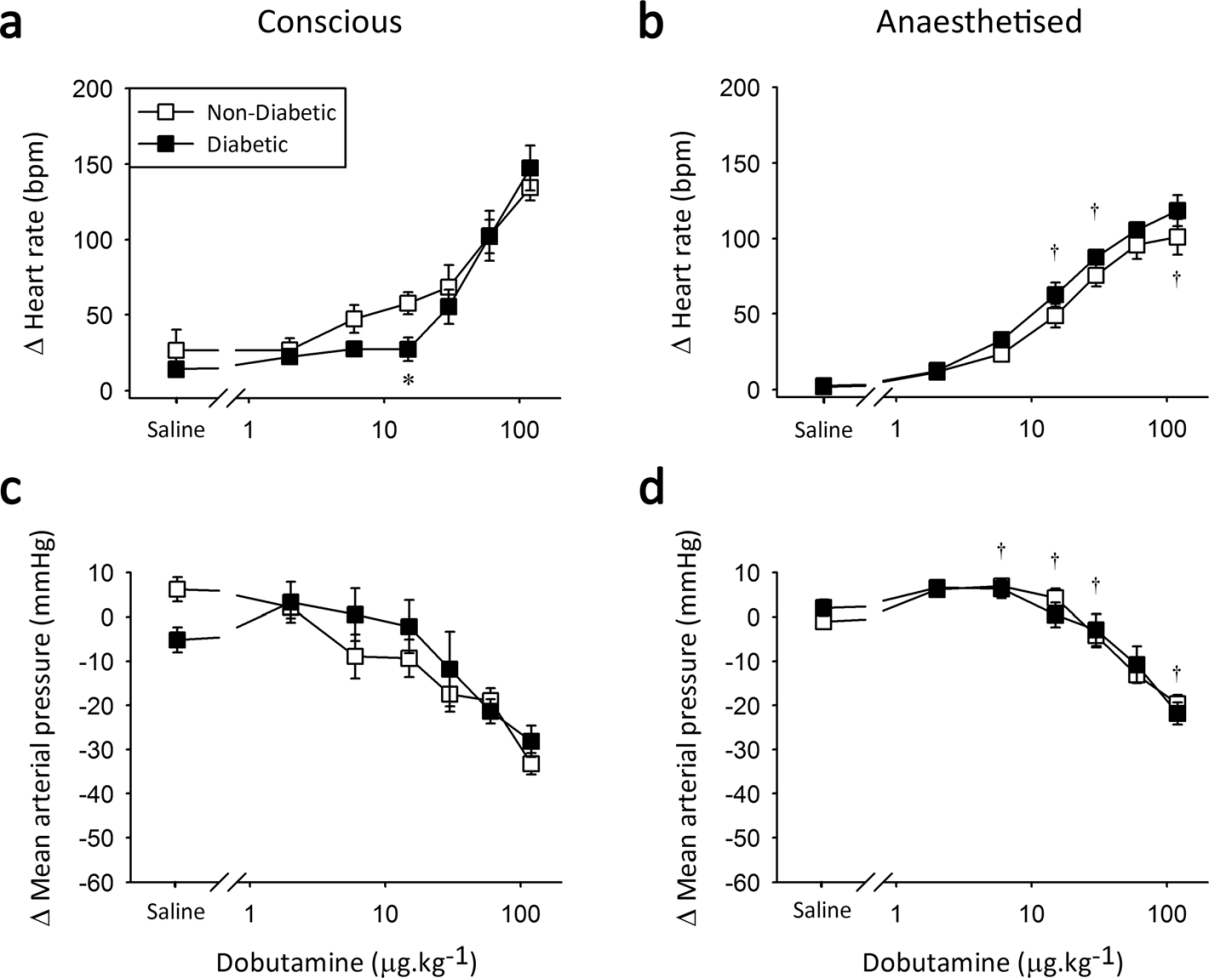
**β-adrenoceptor agonist responses in Zucker Diabetic Fatty rats.** Peak change in heart rate (top) and mean arterial pressure (bottom) in response to 2-120 μg.kg^−1^ dobutamine under conscious conditions **(a and c)** and during isoflurane anaesthesia **(b and d)**. *significantly different from non-diabetic littermate controls and ^†^significantly different from conscious measure, *n* = 7-8, *p* < 0.05, values are means ± SE.

Administration of dobutamine, a non-specific β-AR agonist, primarily elicited a dose-dependent increase in HR in both non-diabetic and type 2 diabetic animals under all conditions (Figure 3). Conscious diabetic rats were less sensitive to dobutamine than their non-diabetic counterparts (Figure 3a), as evidenced by a significantly reduced response to the mid-dose of dobutamine (15 μg.kg^−1^). This dose is within the therapeutic range of approximately 2-20 ug.kg^−1^.min^−1^ [40], making the observed chronotropic difference particularly relevant for the clinical setting. In addition, similar percentage increases in heart rate have been described following injection of 3.2 and 12.2 μg.kg^−1^ dobutamine in healthy human volunteers [41]; indicating that effective doses are equivalent between the species. Isoflurane anaesthesia reduced the maximal β-AR-mediated chronotropic response in non-diabetic animals (Figure 3b). Conversely, in type 2 diabetic animals anaesthesia increased β-AR sensitivity in the mid-range (Figure 3b) compared to the conscious condition, with no change at the maximal dose. Thus, isoflurane normalised β-AR responsiveness in type 2 diabetic rats *in vivo*.

Dobutamine administration also caused a transient decrease in MAP under all conditions, most likely attributable to vasodilation via stimulation of β_2_-ARs in the vasculature (Figure 3c and d). No significant differences in β-AR-mediated vasodilation were observed between non-diabetic and type 2 diabetic animals.

### Baseline characteristics and haemodynamics of obese rats

Following the findings of altered adrenoceptor function and responses to anaesthesia in type 2 diabetic animals *in vivo*, we aimed to determine whether these disturbances were specifically attributable to hyperglycaemia or a general feature of obesity and the metabolic syndrome.

Zucker obese rats exhibited markedly higher body weights than their lean littermates, with an average 60% increase, accompanied by a large increase in epididymal fat pad weight (Table 1). Cardiac hypertrophy became evident in obese animals when heart weight was adjusted to tibia length as a proxy for body size, despite no significant differences in tibia length (data not shown). Metabolic assessment showed hyperinsulinemia with normal glucose levels in obese animals, indicating insulin resistance.

Similar to previous reports [18,42], obese animals displayed significantly increased MAP under both conscious and anaesthetised conditions (Table 1).

### α-adrenergic sensitivity is increased in conscious and anaesthetised obese rats

**Figure 4:**
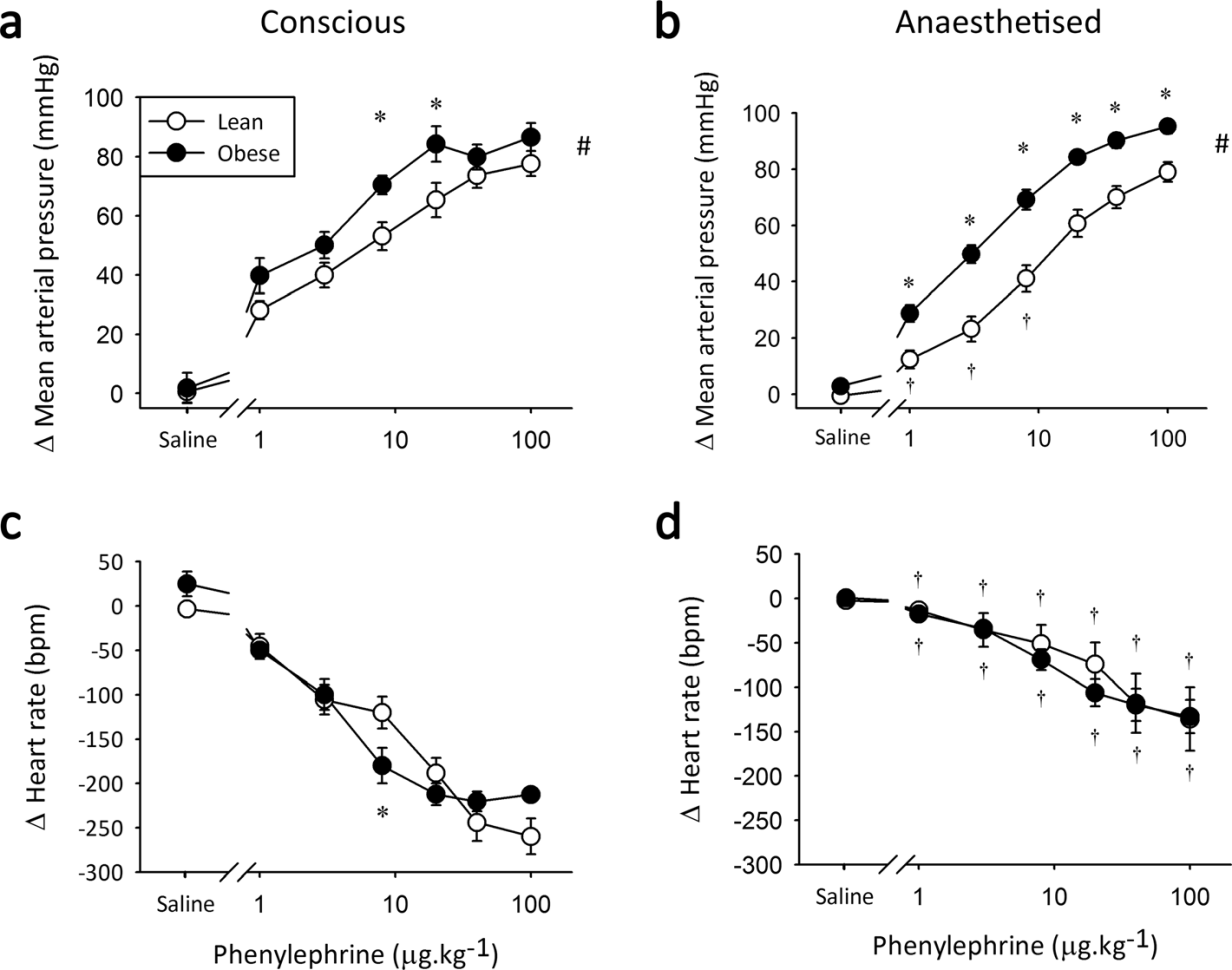
**α-adrenoceptor agonist responses in Zucker Obese rats.** Peak change in mean arterial pressure (top) and heart rate (bottom) in response to 1–100 μg.kg^−1^ phenylephrine under conscious conditions **(a and c)** and during isoflurane anaesthesia **(b and d)**. *significantly different from lean littermate controls, ^†^significantly different from conscious measure and # significant overall group difference, *n* = 8, *p* < 0.05, values are means ± SE.

Conscious obese rats showed a larger increase in MAP with α-AR stimulation than their lean littermates (Figure 4a), suggesting increased α-AR sensitivity in obesity *in vivo*. Isoflurane anaesthesia reduced the MAP response to low doses of PE in lean, but not obese, animals (Figure 4b), exacerbating the differential α-AR-mediated vasoconstriction.

Under conscious conditions, obese rats exhibited increased baroreflex-mediated bradycardia in response to 8 μg.kg^−1^ PE (Figure 4c). However, with a greater corresponding increase in MAP (Figure 4a), this represents an unchanged baroreflex sensitivity (lean −2.3 ± 0.3 vs. obese −2.5 ± 0.2 bpm.mmHg^−1^; *NS*). Conscious obese rats also showed a trend toward reduced maximal bradycardia (Figure 4c; *p* = 0.052), similar to that seen in type 2 diabetic animals. Baroreflex-mediated bradycardia was generally reduced during anaesthesia (Figure 4d), as seen in the ZDF rats, removing potential obesity-derived differences.

### β-adrenergic sensitivity is reduced in conscious obese rats

**Figure 5:**
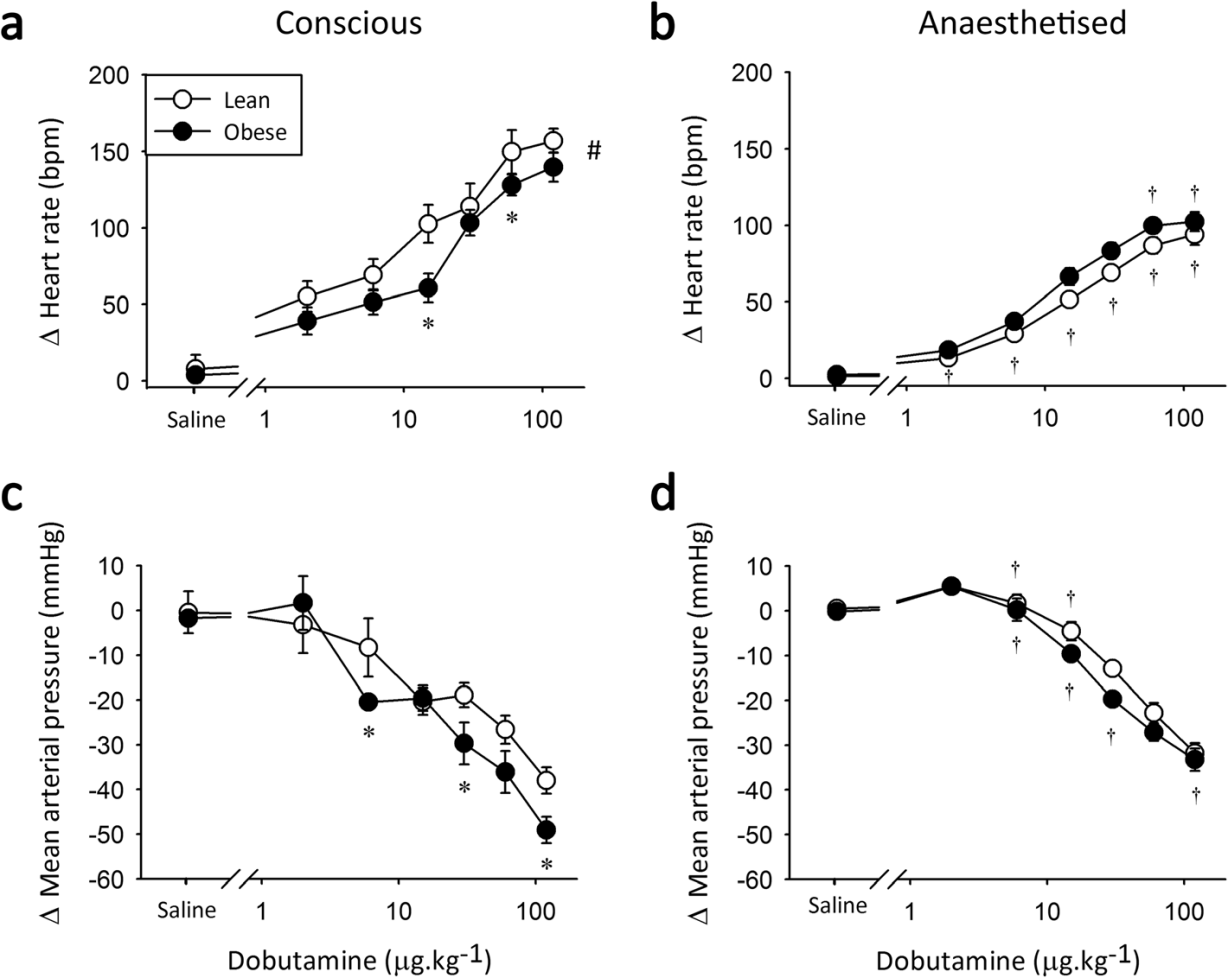
**β-adrenoceptor agonist responses in Zucker Obese rats.** Peak change in heart rate (top) and mean arterial pressure (bottom) in response to 2-120 μg.kg^−1^ dobutamine under conscious conditions **(a and c)** and during isoflurane anaesthesia **(b and d)**. *significantly different from lean littermate controls, ^†^significantly different from conscious measure and # significant overall group difference, *n* = 7-9, *p* < 0.05, values are means ± SE.

Conscious obese rats displayed a reduced β-AR response following dobutamine administration compared to their lean counterparts (Figure 5a), an effect most pronounced at the mid-dose (15 μg.kg^−1^) similar to type 2 diabetic rats. Isoflurane anaesthesia significantly reduced the maximal β-AR-mediated chronotropic response in both groups (Figure 5b), being particularly effective in lean animals where the response was reduced across the entire dose curve. The general reduction in β-AR response during anaesthesia eliminated sensitivity differences between the obese animals and their controls.

Dobutamine-mediated vasodilation was slightly greater in obese than lean rats, with variable significance across the dose–response curve (Figure 5c). Under anaesthesia the differences between lean and obese animals were absent (Figure 5d).

### α-adrenergic responses are prolonged by anaesthesia and type 2 diabetes or obesity

**Figure 6:**
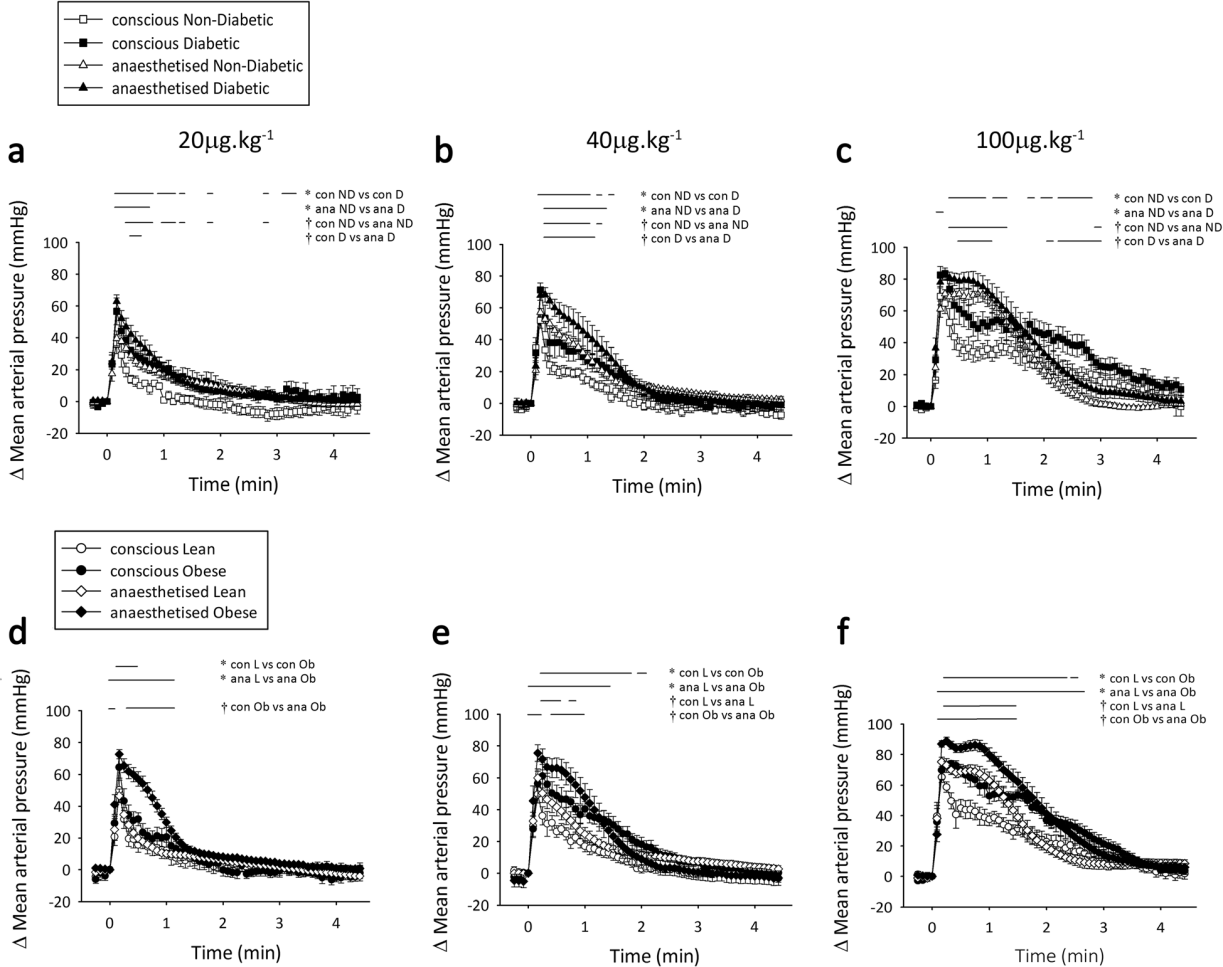
**Time courses of α-adrenoceptor stimulation.** Change in mean arterial pressure in response to 20 **(a and d)**, 40 **(b and e)** and 100 **(c and f)** μg.kg^−1^ phenylephrine in Zucker Diabetic Fatty **(a, b and c)** and Zucker Obese **(d, e and f)** rats. Statistical significance is indicated by the bars above each graph; con: conscious, ana: anaesthetised, ND: non-diabetic, D: diabetic, L: lean, Ob: obese. *n* = 7-9, *p* < 0.05, values are means ± SE.

The interesting observation that the differential α-AR responsiveness in type 2 diabetes and obesity *in vivo* was exacerbated by anaesthesia was further considered with respect to the temporal dynamics of the vasoconstriction. With the rapid effects of PE, no change in time to peak response or time to return to plateau of note was apparent between any groups or conditions. However, observation of the full response time courses indicated significant effects of both anaesthesia and diabetes to prolong the elevated MAP following α-AR stimulation (Figure 6a-c). Similar, and more pronounced, prolongation of the α-AR effects was observed in obese animals (Figure 6d-f). A significant interaction between anaesthesia and obesity was particularly apparent following administration of 20 μg.kg^−1^ PE (Figure 6d). Isoflurane anaesthesia did not prolong the MAP response to equivalent α-AR stimulation in lean animals. Increasing the α-AR agonist concentration uncovered independent, but additive, effects of obesity and anaesthesia to exacerbate α-AR-mediated vasoconstriction (Figure 6e and f). Notably, anaesthesia appeared to primarily exacerbate the MAP response at the early to mid-time points, while obesity prolonged MAP elevation in the later stages of the response.

The time taken to reach peak β-AR HR response was similar in all conscious animals (non-diabetic 31 ± 8 vs. diabetic 48 ± 9 s, lean 34 ± 4 vs. obese 38 ± 5 s at 120 μg.kg^−1^; *NS*). However, during anaesthesia the time to peak response was significantly prolonged in control animals compared to their diseased littermates (non-diabetic 68 ± 6 vs. diabetic 47 ± 3 s, lean 66 ± 5 vs. obese 39 ± 4 s at 120 μg.kg^−1^; *p* < 0.05), despite similar peak values (Figures 3b and 5b). Moreover, the time required for HR to return to plateau from peak dobutamine response was slightly faster in conscious type 2 diabetic animals at the maximal dose (non-diabetic 177 ± 41 vs. diabetic 94 ± 19 s; *p* < 0.05). Isoflurane non-selectively slowed this HR recovery time to plateau. Therefore, alterations in the dynamics of β-AR function in type 2 diabetes and obesity, particularly during anaesthesia, may be reflected more by a briefer, rather than smaller, response.

### Anaesthesia differentially affects adrenergic responses in non-diabetic and type 2 diabetic rats

**Figure 7:**
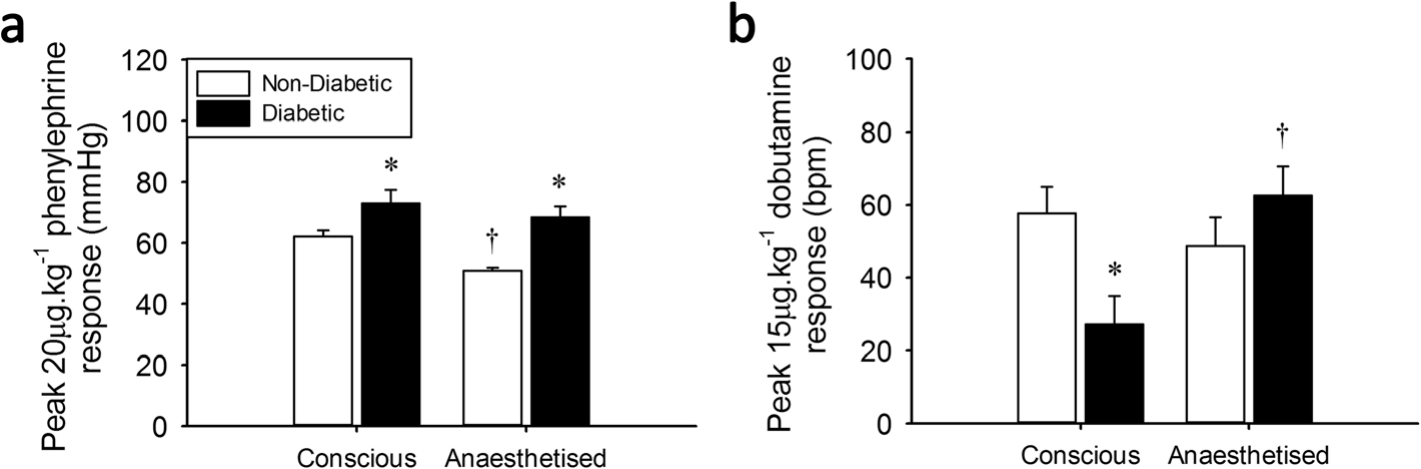
**Summary of α- and β-adrenoceptor function in conscious and anaesthetised Zucker Diabetic Fatty rats.** Peak responses to mid-dose phenylephrine **(a)** or dobutamine **(b)**. *significantly different from lean littermate controls, ^†^significantly different from conscious measure, *n* = 7-9, *p* < 0.05, values are means ± SE.

The broad findings of this study can be summarised in the peak responses to mid-doses of PE (20 μg.kg^−1^) and dobutamine (15 μg.kg^−1^). Conscious type 2 diabetic and obese rats exhibited significantly increased α-adrenergic and reduced β-adrenergic sensitivity. Isoflurane anaesthesia decreased α-adrenergic responsiveness in non-diabetic but not in type 2 diabetic animals, while it increased β-adrenergic responses in type 2 diabetic rats with no effect on control responses (Figure 7). Anaesthetised obese rats similarly exhibited maintenance of the increased α-AR sensitivity, and normalisation of the β-adrenergic responses due to a reduction in the control animals only (data not shown). Thus, anaesthetised type 2 diabetic and obese animals experience a significantly accentuated adrenergic load.

## Discussion

This is the first study to determine the interaction between α- and β-adrenergic function and isoflurane anaesthesia *in vivo* under physiological conscious and anaesthetised conditions. This conscious-anaesthesia approach reveals augmented vascular α-AR sensitivity and reduced cardiac chronotropic β-AR sensitivity in conscious free-moving type 2 diabetic and obese animals. Moreover, isoflurane anaesthesia exacerbated the increased vascular α-AR sensitivity in both disease models, while normalising the chronotropic β-AR responses in obese rats and surprisingly increasing the chronotropic β-AR sensitivity in type 2 diabetic rats. These results show that chronic metabolic stress, such as during type 2 diabetes and obesity, alters α- and β-adrenoceptor function *in vivo*, its dynamics and the interaction with anaesthesia; reducing the haemodynamic capacity of the cardiovascular system to compensate during times of acute stress. Furthermore, the differential α- and β-adrenergic responses described in type 2 diabetes and obesity during isoflurane anaesthesia emphasise the importance of examining pharmacological effects under physiological conditions.

Several studies have found increased α-AR vascular reactivity in isolated and anaesthetised preparations from both type 1 and type 2 diabetic (20, 30, 38) and obese models (28, 29, 37). Alternatively, some studies reported unchanged [18,21] or reduced [43,44] vascular α-AR responses. However use of low phenylephrine doses that also failed to discern a difference in our study [18] or nonspecific α-AR stimulation with noradrenaline [43,44], may explain these differences. The present study shows augmented vascular α-AR responsiveness, in magnitude and in duration, in conscious type 2 diabetic and obese animals, at clinical doses of phenylephrine (32).

The reduced cardiac β-AR sensitivity observed aligns with and extends literature reports. During the metabolically compromised state of diabetes, it is suggested that the sympathetic drive to the heart is increased [4,7], eventually desensitising the adrenergic control of the heart and reducing its function [45]. Reduced β-AR activity has been described in both isolated heart [14-16,46] and in anaesthetised *in vivo* preparations [12] of streptozotocin-induced type 1 diabetes. Furthermore, we have recently described the loss of β-AR responsiveness with type 2 diabetes in human right atrial cardiac muscles [17]. Similar findings of reduced β-AR function have been derived from heart tissue from [19,20] and a single report in conscious [18] obese rodents. However, the present study provides the first evidence of increased and prolonged vascular α-AR sensitivity and reduced chronotropic β-AR sensitivity in well-developed models of both type 2 diabetes and obesity *in vivo* under conscious conditions.

While the mechanisms underlying the altered AR function are poorly understood, and determination was not the aim of this study, changes in AR expression have been implicated. Elevated cardiovascular α_1_-AR expression has been described in various models of the metabolic syndrome [46-48], although decreased α-AR density has also been reported [48,49]. This α_1_-AR variability is proposed to be attributable to disease duration [24,49], with a biphasic expression pattern providing a compensatory response to the progressive α-AR sensitisation [48,49]. However, during ganglionic blockade α-AR responsiveness was found to be unchanged, indicating that the effect may originate downstream [21]. Decreased expression of β_1_-ARs was shown in myocardium of type 1 diabetic rodent models [11,13,50]. However, we found unchanged β_1_-AR expression in right atrial tissue from type 2 diabetic patients [17]. Similarly, Carroll *et al*. [42] found unchanged overall β-AR density in obese rabbit ventricles, and similar to α-ARs, suggested that defective β-AR function in obesity may originate downstream of the receptors themselves. Moreover, we have recently shown that the haemodynamic effects observed using our technique are not due to the potential confounders of volume or injection stress in conscious animals [34]. Thus, further research, particularly into expression and function of α-AR in the vasculature and β-AR in the sinoatrial node, is warranted to explain these haemodynamic and chronotropic differences.

Much of the previous experimental evidence for α- and β-AR-dysfunction has been derived from models of type 1 diabetes, and conducted in anaesthetised or isolated heart or vessel preparations. Therefore, this study, using improved conscious *in vivo* techniques, confirms the relevance of these α- and β-AR-dysfunctions in type 2 diabetes and obesity under physiological conditions and provides valuable new insights. Additionally, the paucity of suitable data in conscious animals has made it difficult to elucidate the effects of anaesthetics per se, and few investigations have addressed this issue. Amour *et al.* [16] showed in an interesting study that halogenated anaesthetics, including isoflurane, increased both α-AR and β-AR inotropic responses in isolated hearts. This is supported by our observation of enhanced β-AR-generated HR responses in type 2 diabetic animals, but not by the absence of isoflurane-potentiated β-AR chronotropic responses in non-diabetic rats or the reduced α-AR sensitivity. This indicates the importance of *in vivo* investigations, including neural, hormonal and vascular feedbacks, which are most likely geared to compensate for excessive fluctuations in the cardiovascular system.

It is unclear how defects exclusively at the α- and β-ARs themselves would directly interact with anaesthesia. Likewise, simple interruption of central autonomic signalling by anaesthetics would be unlikely to selectively interact with α-AR function. The phenylephrine-generated baroreflex-induced changes in HR were overall markedly depressed under isoflurane. Thus, it could be speculated that isoflurane prevents appropriate central control of sympathetic withdrawal during MAP elevation in the vasculature causing an anaesthetic-mediated exacerbation of α-AR sensitisation in type 2 diabetic and obese rats.

By utilising models of type 2 diabetes and obesity with a common underlying defect in leptin signalling, we were able to directly address the influence of hyperglycaemia. The observed alterations in α- and β-AR function were broadly similar between type 2 diabetic and obese rats. Thus, these impairments are likely to be a function of the general metabolic syndrome components of overweight and insulin resistance, rather than the hyperglycaemic phenotype of diabetes; in agreement with previous findings that α- and β-AR dysfunction is not tied to the development of type 2 diabetes [51]. Furthermore, as the effects were more severe in morbidly obese rats, and less pronounced in the overweight type 2 diabetic animals, it appears that obesity may be the primary causal defect.

Regardless of the cause of exacerbated metabolic α- and β-AR dysfunction, differences in α- and β-AR responsiveness (in magnitude and duration) remain an important consideration in this large patient group. During chronic metabolic stress, such as obesity and type 2 diabetes, sympathetic drive to the heart is suggested to be increased [4,7]; which may be partially offset by the observed reduction in β-AR sensitivity. Enhanced and prolonged α-AR responsiveness and the resultant chronic excess cardiovascular pressure could lead to cardiac complications, including increased afterload, cardiac remodelling and hypertrophy [52]. During anaesthesia, the dual effects of exacerbating differences in α-AR response and normalising β-AR function will likely act in concert in type 2 diabetes and obesity to further increase cardiovascular stress. This indicates that chronic metabolic stress limits the capacity of the cardiovascular system to respond when challenged by acute stressors such as anaesthesia. In particular, the interaction between metabolic syndrome and anaesthesia to exaggerate phenylephrine-mediated elevations in MAP suggests that type 2 diabetic and obese patients may be exposed to increased pharmacological stress during surgery.

## Conclusion

In summary, this study is the first to determine the haemodynamic consequences of altered α- and β-AR function in conscious type 2 diabetic and obese animals, and the interaction between adrenergic dysfunction and isoflurane anaesthesia *in vivo*. Under conscious physiological conditions, type 2 diabetic and obese rats exhibited increased α-AR and decreased β-AR responses. Meanwhile, during anaesthesia, enhanced α-AR sensitivity and normalised β-AR function may further impair cardiovascular function in type 2 diabetes and obesity.
